# Screening and predicting progression from high-risk mild cognitive impairment to Alzheimer’s disease

**DOI:** 10.1038/s41598-021-96914-3

**Published:** 2021-09-02

**Authors:** Xiao-Yan Ge, Kai Cui, Long Liu, Yao Qin, Jing Cui, Hong-Juan Han, Yan-Hong Luo, Hong-Mei Yu

**Affiliations:** 1 Department of Health Statistics, School of Public Health, Shanxi Medical University, 56 XinJian South Road, Taiyuan, China; 2 Department of Health Statistics, School of Public Health, Jinzhou Medical University, 40 SongPo Road, Jinzhou, China; 3 Department of Mathematics, School of Basic Medical Sciences, Shanxi Medical University, Taiyuan, China; 4 Shanxi Provincial Key Laboratory of Major Diseases Risk Assessment, 56 XinJian South Road, Taiyuan, China

**Keywords:** Diseases, Medical research, Risk factors

## Abstract

Individuals with mild cognitive impairment (MCI) are clinically heterogeneous, with different risks of progression to Alzheimer’s disease. Regular follow-up and examination may be time-consuming and costly, especially for MRI and PET. Therefore, it is necessary to identify a more precise MRI population. In this study, a two-stage screening frame was proposed for evaluating the predictive utility of additional MRI measurements among high-risk MCI subjects. In the first stage, the K-means cluster was performed for trajectory-template based on two clinical assessments. In the second stage, high-risk individuals were filtered out and imputed into prognosis models with varying strategies. As a result, the ADAS-13 was more sensitive for filtering out high-risk individuals among patients with MCI. The optimal model included a change rate of clinical assessments and three neuroimaging measurements and was significantly associated with a net reclassification improvement (NRI) of 0.246 (95% CI 0.021, 0.848) and integrated discrimination improvement (IDI) of 0.090 (95% CI − 0.062, 0.170). The ADAS-13 longitudinal models had the best discrimination performance (Optimism-corrected concordance index = 0.830), as validated by the bootstrap method. Considering the limited medical and financial resources, our findings recommend follow-up MRI examination 1 year after identification for high-risk individuals, while regular clinical assessments for low-risk individuals.

## Introduction

Alzheimer’s disease (AD) is the most common cause of dementia in developing countries and is expected to affect 1 in 85 people worldwide by the year 2050^1^. Mild cognitive impairment (MCI) is a transitional stage between normal cognition and dementia, and MCI patients are commonly enrolled as the target population for evaluating prognosis and early intervention for dementia^2,3^. Moreover, individuals with MCI are clinically heterogeneous, with different risks of progression to AD^4^. Therefore, identifying patients with MCI who are at risk of progression to AD and improving the prognosis of MCI is of vital importance in the personalised clinical management of AD.

Existing studies have used multimodal information, including neurocognitive, magnetic resonance imaging (MRI), CSF-based, and positron emission tomography (PET) markers for binary classification modeling^5–7^. Although these studies have shown high classification accuracy, some approaches are often unavailable in the primary clinical setting, considering the cost and invasiveness of the procedures. Numerous convenient medical checks, such as neuropsychological tests, are preferred in primary screening^8–10^. Advanced examination technologies should be considered only when they are necessary for more precise diagnosis; otherwise, their value in the predictive model may be offset by their inconvenience. To maximise the value of these advanced detection methods and to reduce the burden on patients, it is necessary to focus on those at high risk of developing AD^11–14^.

Additionally, many studies predicted the probability of conversion from MCI to AD as a binary response at a fixed time point (i.e., 3 years or 5 years), which did not consider the time interval during the conversion. Some studies used Cox regression models to investigate the time of the development from MCI to AD during follow-up^15–17^; however, most of these studies evaluated the predictive effect of single or combined prognostic markers only at the baseline^18^. In actual clinical practice, we often collect follow-up information on prognostic markers, such as the cognitive assessment and MRI examinations. It is necessary to make full use of these follow-up information to predict the progression of MCI.

In the current study, we constructed a two-stage screening frame for individuals with MCI and evaluated the prognostic value of MRI in high-risk subjects with MCI. In stage I, we built the Alzheimer Disease Assessment Scale Cognitive 13 items (ADAS-13) and Mini Mental State Examination (MMSE) trajectory-templates of cognitive decline. In stage II, two trajectory-templates were used to filter out high-risk MCI subjects. Then, these subjects were inputted into the prognosis models which were constructed with different strategies for the two groups.

## Results

### Demographics

Table 1 shows the baseline demographic characteristics (age, gender, marital status, and education), genetic information (ApoEε4), clinical assessment scores (ADAS-13_bl, MMSE_bl, and the baseline of Clinical Dementia Rating Sum of Boxes (CDRSB_bl)), and trajectory label (ADAS-13 trajectory label, MMSE trajectory label) of subjects in two stages. Of the 85 subjects in stage I (trajectory dataset), 35 (41.2%) progressed to AD over the 3 years of follow-up, with the mean time to onset of AD being 20.4 ± 8.5 months (range 6.0–36.0 months). In stage II, 80 MCI subjects (21.4%) progressed to AD over 3 years, with the mean time to onset of AD being 20.4 ± 10.7 months (range 6.0–36.0 months).

**Table 1:** Baseline characteristics of subjects in two stages.

Characteristics	Mean (SD) or N (%)	
	Stage I: ADNI-1 (N = 85)	Stage II: ADNI GO/2 (N = 374)
Age (year)	74.2 (6.9)	71.2 (7.4)
**Gender**		
Male	61 (71.8)	206 (55.1)
Female	24 (28.2)	168 (44.9)
**Marital status**		
Married	73 (85.9)	278 (74.3)
Divorced	2 (2.4)	43 (11.5)
Others	10 (11.8)	53 (14.2)
**Education**		
Medium	0 (0.0)	2 (0.5)
High	85 (100.0)	372 (99.5)
**ApoEε4**		
Absent	36 (42.4)	199 (53.2)
Present	49 (57.7)	175 (46.8)
**ADAS-13 Trajectory Label**		
Low-risk	44 (51.8)	264 (70.6)
High-risk	41 (48.2)	110 (29.4)
**MMSE Trajectory Label**		
High-risk	50 (58.8)	92 (24.6)
Low-risk	35 (41.2)	282 (75.4)
ADAS-13_bl	30.3 (17.0)	14.5 (6.4)
MMSE_bl	27.5 (1.6)	28.1 (1.7)
CDRSB_bl	1.6 (0.9)	1.4 (0.9)
Total	85	374

ADAS-13, Alzheimer Disease Assessment Scale Cognitive 13 items; MMSE, mini mental state examination; ADAS-13_bl, Alzheimer Disease Assessment Scale Cognitive 13 items at Baseline; MMSE_bl, mini mental state examination at Baseline; CDRSB_bl, Clinical Dementia Rating Sum of Boxes at Baseline; SD, standard deviation.

### Stage I: Trajectory modelling

**Figure 1:**
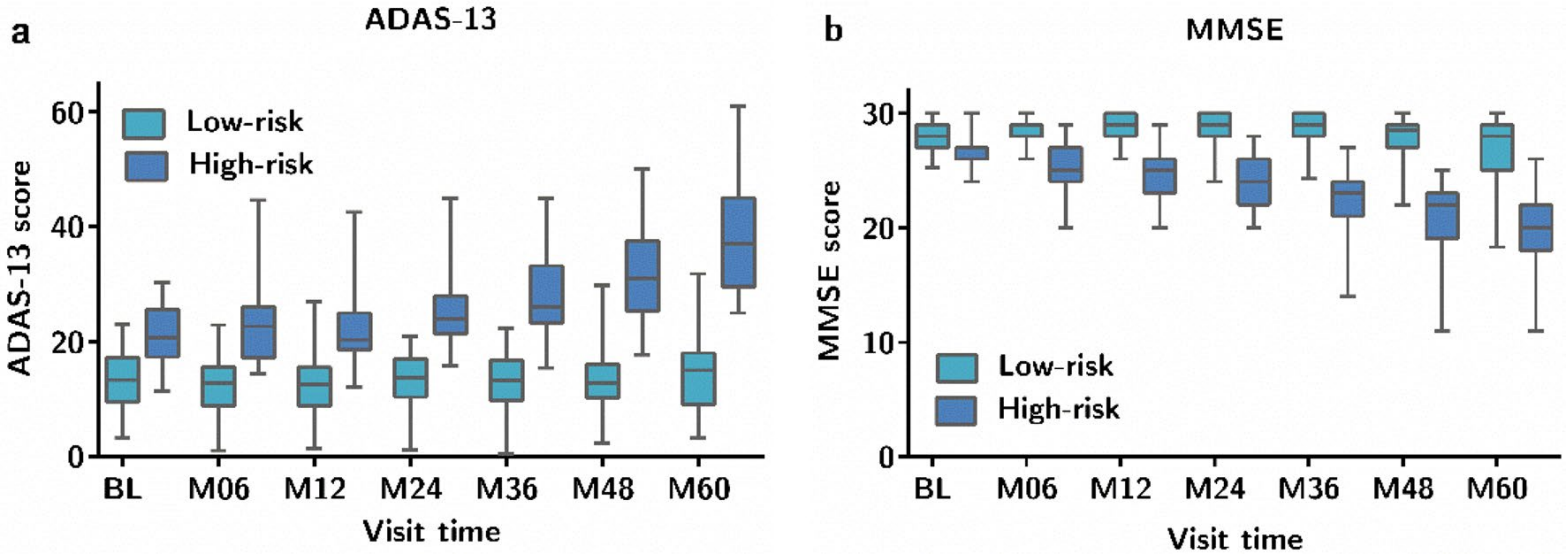
Clinical score distributions at each timepoint for the different trajectories derived from the K-means clustering (N = 85). The mean scores at each timepoint are used to build a template for each trajectory class. (**a**) Trajectory based on ADAS-13; (**b**) trajectory based on MMSE.

K-means clustering based on ADAS-13 and MMSE separately generated two clusters, namely high-risk and low-risk (Fig. 1). Higher ADAS-13 scores and lower MMSE scores resulted in a higher risk of cognitive decline from MCI to AD. We used trajectory-templates to assign a trajectory label to the 374 patients according to the Euclidean proximity computed from all available time points.

### Stage II: Performance of the MCI prognosis models

The subjects labelled with high-risk MCI were filtered out and may be prone to developing AD. Table 2 summarises the baseline characteristics and six change rate predictors in the first year of the high-risk MCI patients stratified by dementia status at 3 years. In the ADAS-13 predictive datasets, the groups with and without dementia differed significantly with respect to CDRSB_bl, $\Delta$CDRSB, $\Delta$Hippocampus, $\Delta$WholeBrain, but had similar distributions of age, sex, marital status, education, ApoEε4, MMSE_bl, ADAS-13_bl, Hippocampus_bl, WholeBrain_bl, Entorhinal_bl, $\Delta$MMSE, $\Delta$ADAS-13, and $\Delta$Entorhinal. In the MMSE predictive dataset, the groups with and without dementia were significantly different with respect to ADAS-13_bl and $\Delta$MMSE, while the other results were consistent with those of the ADAS-13 predictive datasets.

**Table 2:** Predictors of high-risk patients with MCI based on ADAS-13 (N = 110) and MMSE (N = 92) predictive datasets, stratified by dementia status over 3 years of follow-up.

Characteristics	ADAS-13 predictive dataset (N = 110) Mean (SD) or N (%)		MMSE predictive dataset (N = 92) Mean (SD) or N (%)	
	No dementia (N = 44)	Dementia (N = 66)	No dementia (N = 35)	Dementia (N = 57)
Age (year)	74.3 (1.9)	71.9 (7.4)	75.1 (6.8)	72.6 (6.9)
**Gender**				
Male	27 (61.4)	35 (53.0)	22 (63.9)	33 (57.9)
Female	17 (38.6)	31 (47.0)	13 (37.1)	24 (42.1)
**Marital status**				
Married	34 (77.3)	53 (80.3)	28 (80.0)	47 (82.5)
Divorced	2 (4.5)	4 (6.1)	2 (5.7)	4 (7.0)
Others	8 (18.2)	9 (13.6)	5 (14.3)	6 (10.5)
**Education**				
Medium	0 (0.0)	0 (0.0)	0 (0.0)	0 (0.0)
High	44 (100.0)	66 (100.0)	35 (100.0)	57 (100.0)
**ApoEε4**				
Absent	21 (47.7)	21 (31.8)	13 (37.1)	17 (29.8)
Present	23 (52.3)	45 (68.2)	22 (62.9)	40 (70.2)
ADAS-13_bl	20.7 (4.0)	22.4 (5.4)	19.2 (5.0)	22.2 (6.0)*
MMSE_bl	27.6 (1.7)	27.1 (1.7)	26.5 (1.5)	26.7 (1.6)
CDRSB_bl	1.5 (0.8)	2.2(1.0)**	1.5 (0.8)	2.2 (0.9)*
Hippocampus_bl (cm^3^)	6.4 (1.0)	6.4 (1.0)	6.5 (1.0)	6.4 (1.0)
WholeBrain_bl (cm^3^)	1042.3(105.1)	1048.3(114.6)	1033.2(100.8)	1052.0 116.6)
Entorhinal_bl (cm^3^)	3.5 (0.6)	3.3 (0.7)	3.5 (0.6)	3.3 (0.7)
$\Delta$ADAS-13	− 0.2 (4.9)	2.4 (4.2)	− 0.1 (5.9)	2.7 (4.6)
$\Delta$MMSE	− 1.3 (2.1)	− 1.5 (1.9)	− 1.1 (1.8)	− 1.6 (1.9)*
$\Delta$CDRSB	0.3 (0.8)	1.0(1.1) **	0.4 (0.9)	1.0 (1.1)*
$\Delta$Hippocampus (cm^3^/year)	− 0.1 (0.3)	− 0.3 (0.2) *	− 0.2 (0.2)	− 0.3 (0.2)*
$\Delta$WholeBrain (cm^3^/year)	− 5.4 (14.5)	− 15.6 (10.8)**	− 6.6 (15.8)	− 15.8(11.6)*
$\Delta$Entorhinal (cm^3^/year)	− 0.1 (0.3)	− 0.2 (0.3)	− 0.1 (0.3)	− 0.2 (0.3)

ADAS-13_bl, Alzheimer Disease Assessment Scale Cognitive 13 items at Baseline; MMSE_bl, Mini mental state examination at Baseline; CDRSB_bl, Clinical Dementia Rating Sum of Boxes at Baseline; ∆ADAS-13, (ADAS-13_M12 − ADAS-13_bl)/1 year; ∆MMSE, (MMSE_M12 − MMSE_bl)/1 year; ∆CDRSB, (CDRSB_M12 − CDRSB_bl)/1 year; Hippocampus_bl, The Total Volumes of The Hippocampus at Baseline; WholeBrain_bl, Whole Brain at Baseline; Entorhinal_bl, Entorhinal Cortex at Baseline; ∆Hippocampus, (Hippocampus _M12 − Hippocampus _bl)/1 year; ∆WholeBrain, (WholeBrain _M12 − WholeBrain _bl)/1 year; ∆Entorhinal, (Entorhinal _M12 − Entorhinal _bl)/1 year; SD, Standard deviation.

**P < 0.001; *P < 0.05.

Cox proportional hazards regression models were developed using all available data under different strategies for the 3-year progression to AD prediction. Five Cox models based on the ADAS-13 (MMSE) prediction dataset are shown in Supplementary Table S1 (Supplementary Table S2), with their hazard ratios and 95% confidence intervals (CIs).

**Figure 2:**
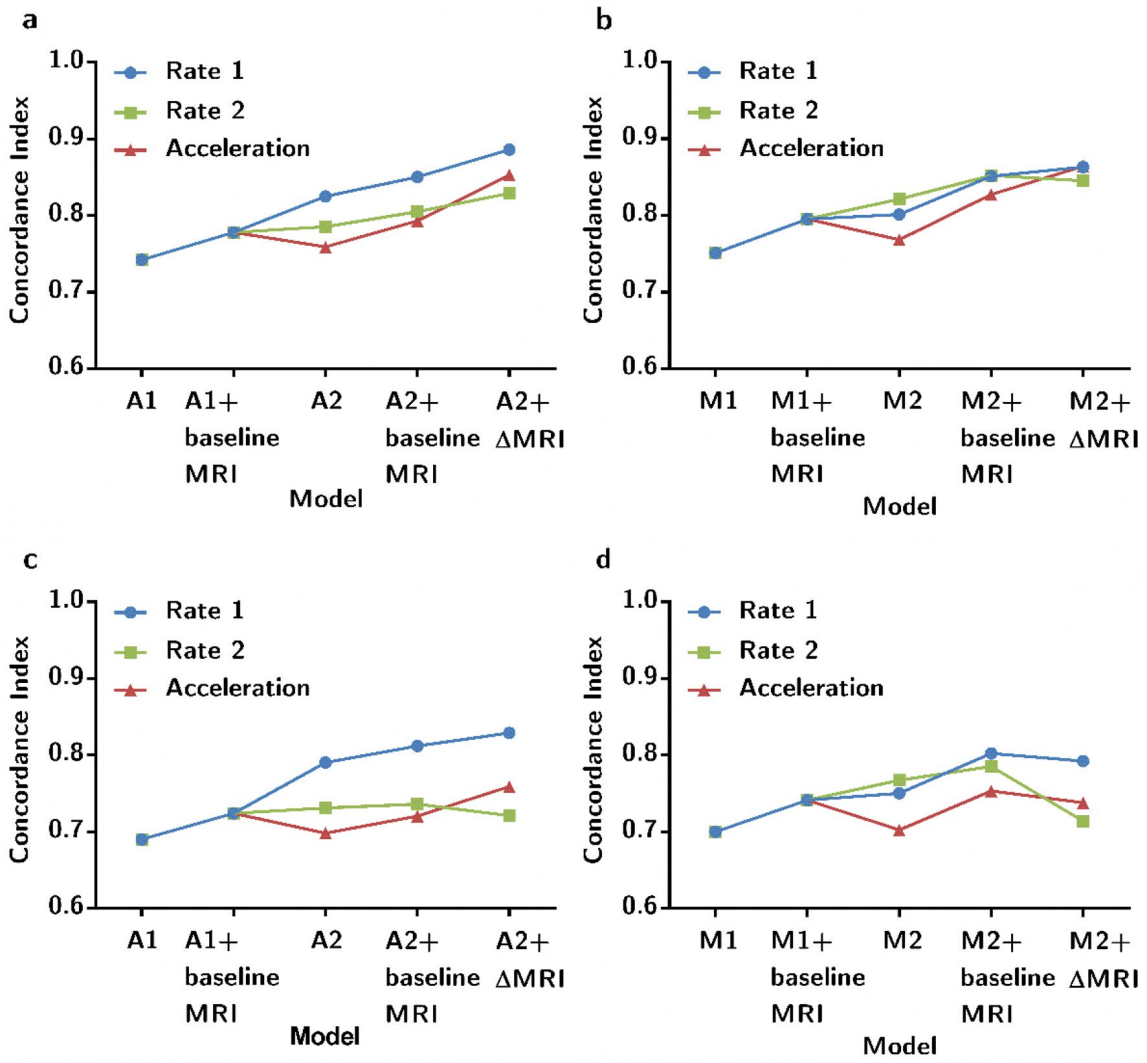
The concordance index (C-index) of models using different strategies to predict progression to dementia within 3 years. (**a**) The concordance index of ADAS-13 predictive dataset (N = 110); (**b**) the concordance index of MMSE predictive dataset (N = 92); (**c**) the concordance index of ADAS-13 predictive dataset by internet validation (bootstrap) (N = 110); (**d**) The concordance index of MMSE predictive dataset by internet validation (bootstrap) (N = 92).

For the ADAS-13 base model, we incorporated seven conventional risk factors into the baseline model A1 but found that only ADAS-13_bl was a significant predictor. The extended model A1+ baseline MRI with the addition of three baseline MRI measurements, and there were five significant predictors including age, marital status, CDRSB_bl, MMSE_bl, and ADAS-13_bl. As shown in Fig. 2, the concordance index (C-index) of model A1+ baseline MRI clearly increased with the inclusion of three baseline MRI measurements. Meanwhile, the NRI was 0.123 (95% CI 0.045, 0.664), indicating that the predictive performance of model A1+ baseline MRI improved by 12.3% compared to that of model A1 (Table 3). The IDI was 0.003, and the confidence interval included zero.

**Table 3:** Performance of prognosis models among high-risk patients with MCI.

Predictive dataset	Prognosis model	NRI (95% CI)	IDI (95% CI)
ADAS-13 (N = 110)	A1+ baseline MRI versus A1	0.123 (0.045, 0.664)*	0.003 (− 0.118, 0.093)
	A2+ baseline MRI versus A2	− 0.062 (− 0.074, 0.391)	0.006 (− 0.172, 0.172)
	A2+ ∆MRI versus A2	0.246 (0.021, 0.848)*	0.090 (− 0.094, 0.209)
MMSE (N = 92)	M1+ baseline MRI versus M1	0.177 (− 0.035, 0.902)	0.008 (− 0.085, 0.087)
	M2+ baseline MRI versus M2	0.201 (− 0.046, 0.685)	0.005 (− 0.148, 0.107)
	M2+ ∆MRI versus M2	0.489 (− 0.132, 0.878)	0.065 (− 0.062, 0.170)

A1, ADAS-13 baseline Prognosis Model; A2, ADAS-13 longitudinal Prognosis Model; M1, MMSE baseline Prognosis Model; M2, MMSE longitudinal Prognosis Model. ADAS-13, Alzheimer Disease Assessment Scale Cognitive 13 items; MMSE, Mini mental state examination; NRI, Net Reclassification Improvement; IDI, Integrated Discrimination Improvement. Values based on the following assumptions: Risk of event = 10%.

*The indices are statistically significant.

For the ADAS-13 longitudinal model, we added three change rates of clinical assessments in the longitudinal model A2, the significant variables were age, CDRSB_bl, ADAS-13_bl, $\Delta$CDRSB, and $\Delta$ADAS-13. A2+ baseline MRI included three baseline MRI imaging measurements based on A2, although these three neuroimaging variables were not significant in predicting the progression of MCI subjects, the discrimination performance of the A2+ baseline MRI was slightly improved as measured by the C-index. Similarly, three additional changes in MRI imaging measurements were incorporated into A2, resulting in six significant predictors in the model A2+ $\Delta$MRI (age, CDRSB_bl, ADAS-13_bl, $\Delta$CDRSB, $\Delta$WholeBrain, and $\Delta$Entorhinal). Figure 2 shows that the C-index of model A2+ $\Delta$MRI was the highest, and it slightly increased with the inclusion of three change values of MRI imaging measurements compared to A2. Meanwhile, the NRI was 0.246 (95% CI 0.021, 0.848), and the IDI was 0.090 (95% CI − 0.094, 0.209). This indicated that the predictive performance of model A2+ $\Delta$MRI improved by 24.6% compared to that of model A2 (Table 3) by NRI. We undertook internal validation by bootstrap, and the longitudinal model A2+ ∆MRI had the best discrimination performance (Optimism-corrected c-index = 0.830) (Supplementary Table S3).

For the MMSE base model, the Cox model was built using a similar strategy. As shown in Fig. 2, the extended model M1+ baseline MRI clearly increased with the inclusion of three baseline MRI measurements. Both the NRI and IDI indices were positive, but the confidence interval included zero.

For the MMSE longitudinal model, the C-index of the extended model M2+ baseline MRI with additional three baseline MRI imaging measurements increased significantly (Fig. 2). Both the NRI and IDI indices were positive, although the confidence interval did not include zero (Table 3). Figure 2 shows that the C-index of model M2+ baseline MRI was the highest. It did not increase with the inclusion of three change rates of MRI measurements in the validation of the data. Meanwhile, the longitudinal model M2+ baseline MRI validated by bootstrap had the best discrimination performance (Optimism-corrected c-index = 0.802) (Supplementary Table S3).

Moreover, to compare the change rate in the first year, we also computed for the cognitive decline rate in the second year and acceleration in the first 2 years (Fig. 2). The Cox model incorporating the change rate in the first year had the best predictive performance, except for M2. A2+ $\Delta$MRI, including the change rates of clinical assessment scores and MRI measurements, showed the best performance of the prognosis model for patients with MCI.

## Discussion

In this study, we constructed and validated a two-stage screening frame for subjects with MCI at an increased risk of developing AD in 3 years.

Previous studies have indicated that clinical assessment tests are excellent at predicting patients with MCI progressing to AD and should be a critical component of identifying AD at the predementia stage^19^. In addition, there was a significant improvement in classifying MCI that were at risk using the duration of follow-up^20^. We enrolled subjects assessed by clinical assessments at seven time points over 5 years to characterise the MCI progression trajectory.

**Figure 3:**
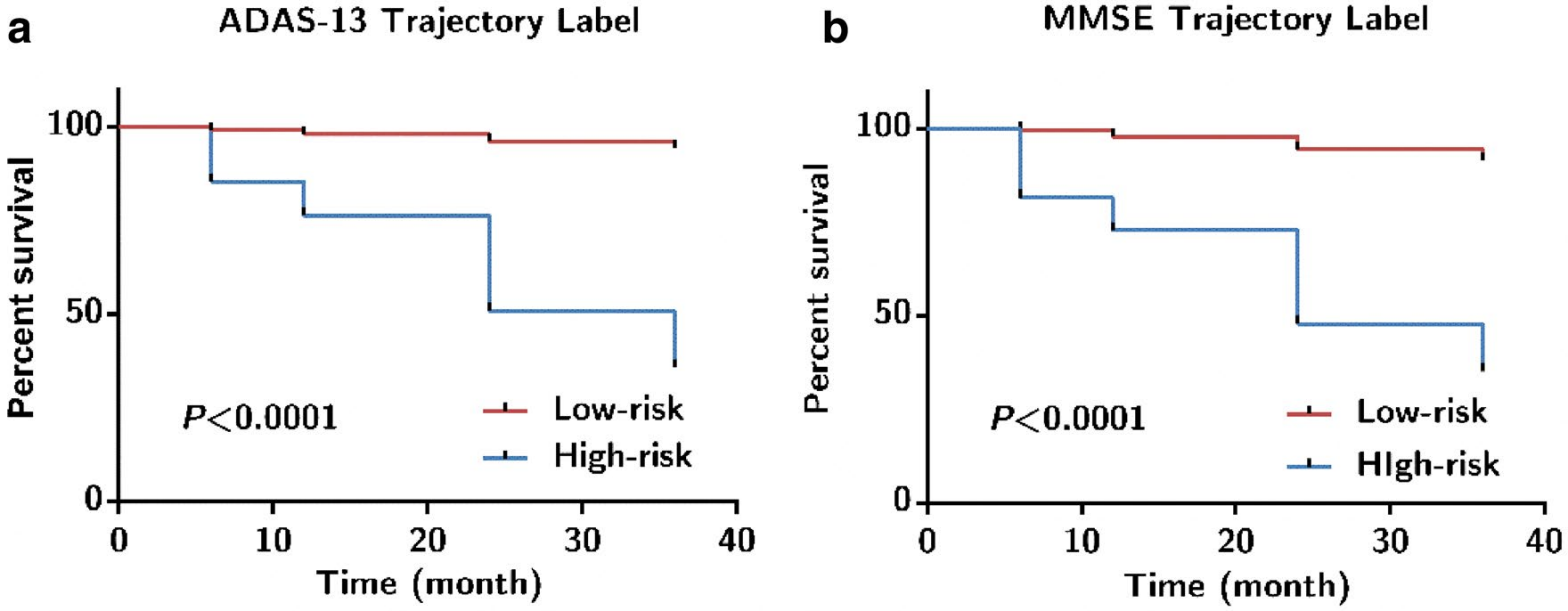
Kaplan–Meier curves for 3-year progression to AD in MCI based on ADAS-13 (N = 110) and MMSE (N = 92) predictive dataset. (**a**) ADAS-13 Trajectory Label; (**b**) MMSE Trajectory Label.

Considering the heterogeneity of MCI progression, we chose two clusters for the ADAS-13 and MMSE. The clustering yielded two groups, namely the high-risk and low-risk group, which reflected the different risks of progression to AD (see Fig. 3). The number of clusters depended not only on the data-driven approach but also on the desired specificity of the trajectory. Higher ADAS-13 scores and lower MMSE scores indicate a higher risk of conversion to AD^21^. Trajectory labels were used to filter out high-risk patients with MCI. Several studies have utilised hierarchical clustering to identify target populations, but they considered trajectory labels as the goal of the predictive task^22–24^. This strategy has three advantages. First, the trajectory labels characterised the progression of MCI without being restricted by strict cut-offs, defining a specific time window. Second, the trajectory-templates could assign a trajectory label for subjects with missing follow-up data^22^; in other words, the technique provides a method for solving the problem of missing time points in a longitudinal study. Third, the trajectory labels can be used to predict the risk of AD onset at an individual level and provide a screening strategy for high-risk patients with MCI^25^. This may have useful clinical implications for more precise disease management and personalised interventions.

Considering the two different progression trajectories of MCI, we filtered out high-risk patients who tended to develop cognitive decline and may progress to AD in the future. Cox models were developed to evaluate the value of additional MRI measurements for a 3-year prognosis model. The results imply that models with additional MRI measurements could improve predictive performance. Therefore, high-risk patients with MCI should be recommended for a follow-up MRI 1 year later. Several studies have evaluated the predictive utility of prognostic markers, but only focused on baseline measurements^15^. One study developed a joint modelling of longitudinal markers and a time-to-event data method to evaluate the effects of longitudinal markers. However, this method only analysed each marker independently^18^. We transformed the longitudinal predictors for change rate and constructed base and longitudinal prognosis models including multiple predictors for different clinical application scenarios.

In the current study, the predictive performance of the baseline model (A1, A1+ baseline MRI) was lower than that of the longitudinal model (A2, A2+ baseline MRI, and A2+ $\Delta$MRI) according to the C-index (see Fig. 2). Similarly, the predictive performance of the baseline model (M1, M1+ baseline MRI) was lower than that of the longitudinal model (M2+ baseline MRI, M2+ $\Delta$MRI) (see Fig. 2). As CDRSB_bl did not satisfy the proportional hazards assumption in models A1, M1, and M2, we translated the continuous variable CDRSB_bl into the categorical variable (CDRSB_bl1) using X-tile software. The stratified Cox models were then performed using CDRSB_bl1 in models A1, M1, and M2 and by marital status in A2.

Overall, the ADAS-13 longitudinal model has better performance than that of MMSE. The results indicate that ADAS-13 is more sensitive in classifying patients with MCI and filtering out high-risk individuals. Furthermore, the A2+ $\Delta$MRI model had the best predictive performance for progression to AD among high-risk patients with MCI. There were six significant variables in A2+ $\Delta$MRI, including age, CDRSB_bl, ADAS-13_bl, $\Delta$CDRSB, $\Delta$WhileBrain, and $\Delta$Entorhinal. We computed the change rate in the first year simply and avoided the longitudinal variables that needed complex technology. Meanwhile, it is helpful to find an interpretable marker in the early stage. In model A2+ $\Delta$MRI, $\Delta$WholeBrain and $\Delta$Entorhinal significantly improved the predictive performance for AD over a 3-year follow-up. Several studies have also shown that atrophy estimates in characteristically vulnerable brain regions, such as the hippocampus and entorhinal cortex, reflect the disease stage and are predictive of progression of MCI to AD^26^.

Here, we propose a two-stage screening framework for MCI subjects facing the risk of AD. The trajectory-template could classify the subjects with MCI into high-risk and low-risk groups at the individual level. When an individual with MCI is predicted to be high-risk, more assessments and additional MRI 1 year later and personalised interventions would be recommended by the clinician. Otherwise, low-risk MCI subjects should be considered for regular clinical assessment. The goal of prognosis models is to provide a simple and accurate tool to forecast the risk of high-risk patients progressing to AD in 3 years^27^. In short, this strategy could facilitate decision-making pertaining to the frequency and monitoring methods that should be conducted in MCI individuals^12,28^. Additionally, it also saves multiple medical resources and reduces the patient’s burden.

This study has some limitations. First, the ADNI cohorts may not represent the general population, and most of the subjects were well-educated. Next, because of the two-stage design of the screening frame, only high-risk patients were filtered out according to the trajectory labels. Because of the small sample size used for prognosis models, we ran the analyses on the entire sample, instead of splitting the sample into training and testing datasets^29^. However, the performance of the prognosis models was evaluated via internal validation (bootstrap method). Bootstrapping is an attractive method for internal validation, and it uses the entire dataset for model development and provides nearly unbiased estimates of predictive accuracy^30^. Whether MRI enhances prediction in other dementia risk models requires further independent validation. Finally, we did not consider the specific money and time saved using this method, but this is our future work.

The key strength of this study is that it is a two-stage screening strategy for MCI patients, which is likely to have clinical utility and save on medical resources. In future studies, we may explore additional MRI clinical application scenarios for different targeting populations.

In conclusion, the study indicated that follow-up MRI examination is recommended 1 year after identification for high-risk patients, and regular clinical cognitive assessments for low-risk MCI patients, especially considering limited medical and financial resources. We believe that this work will further motivate the exploration of multimodal longitudinal prognosis models, which will improve prognostic predictions in MCI.

## Materials and methods

### Datasets and subjects

The data used in this study were downloaded from the Alzheimer’s Disease Neuroimaging Initiative (ADNI) database (http://adni.loni.usc.edu/). The ADNI was launched in 2003, and the primary goal of ADNI was to test whether serial clinical assessments, neuropsychological assessments, MRI, PET, and other biological markers can be combined to measure the progression of MCI and early AD. Detailed information regarding the ADNI study procedures, including participant inclusion and exclusion criteria, can be found at http://www.adni-info.org. As part of the ADNI, subjects with MCI were assessed using the ADAS-13 and MMSE at baseline and at 6, 12, 24, 36, 48, and 60 months. For stage I modelling, 85 ADNI-1 subjects with MCI were included in the trajectory dataset, which was used to build trajectory-templates of cognitive decline. For stage II, 374 subjects with MCI from ADNI GO/2 with two clinical assessments in at least four time points over 5 years were assigned trajectory labels. MCI subjects labelled as high-risk were filtered out and included in the predictive dataset. The ADNI was approved by the institutional review board of each participating site. All participants provided written informed consent. For more information, see http://www.adni-info.org. This study was approved by the ethics committee of Shanxi Medical University. All methods were carried out in accordance with relevant guidelines and regulation. We considered all these subjects as MCI patients in our analysis. The criteria for MCI diagnosis were the same as those defined by Hojjati et al.^31^.

### Measures

In the present study, the potential prognostic factors were clinical assessment and MRI measurements at baseline and their change rate in the first year. The Alzheimer Disease Assessment Scale Cognitive (ADAS-Cog) is a widely used clinical assessment tool for evaluating the cognitive characteristics of AD. The ADAS-13 is the total score of 13 items ranging from 0 to 85, with higher scores indicating poorer cognitive function. The MMSE includes 11 questions with scores ranging from 0 to 30, with lower scores reflecting more severe cognitive impairment^10^. The Clinical Dementia Rating Scale (CDR) is widely used for the detection and severity classification of AD. We usually calculated the Clinical Dementia Rating Sum of Boxes (CDRSB) ranging from 0 to 18, with higher scores indicating more severe dementia^32,33^. Evidence supports the usefulness of CDR in detecting MCI and dementia. CDR should be considered for staging cognitive impairment in at-risk populations^34^. Three structural MRI measurements associated with cognitive decline and dementia, the total volumes of the hippocampus, whole brain, and entorhinal cortex were selected for analysis^29,35^. The details of the MRI protocols used to acquire the image datasets in the ADNI project can be found at http://adni.loni.usc.edu/methods/documents/. Demographic data and apolipoprotein E (ApoE) ε4 allele status (present or absent) were collected at the baseline visit.

### Stage I: Trajectory modelling

The progression trajectory was characterised using multiple-timepoint clinical assessment scores, including the ADAS-13 and MMSE scales^22,36^. The trajectory dataset was used as the input for K-means clustering. The Euclidean distance between the longitudinal clinical assessment score vectors was used as a similarity metric, and Ward’s method was used as a linkage criterion for clustering. The average clinical assessment scores from each cluster at each time point were used as trajectory templates for each class.

### Stage II: Construction and evaluation of the MCI prognosis models

The trajectory template was used to assign a trajectory label for the 374 ADNI-GO/2 MCI subjects. High-risk MCI subjects were filtered out according to the ADAS-13 and MMSE trajectories (n = 110 and 92, respectively). To evaluate the benefit of MRI measurements for predicting the conversion of MCI, we developed 3-year survival models using high-risk patients. The main outcome of the prognosis model was AD that was first diagnosed during follow-up. Multivariate models were calculated using the Cox proportional hazards regression analysis. The proportional hazards assumption was tested in R, and the variable that did not satisfy the assumption was input into the model as a stratified variable. For convenience, the first letter of the ADAS-13 and MMSE was used to distinguish between the two different predictive datasets. The abbreviations are used to represent the different prognosis models. Models A1 and A2 indicate baseline and longitudinal models for ADAS-13, while Models M1 and M2 indicate baseline and longitudinal models for MMSE. The extended models were renamed according to the increased variables compared to the baseline and longitudinal models (Table 4). Considering the longitudinal nature, we computed the change rate of clinical assessment scores ($\Delta$ADAS-13, $\Delta$MMSE, and $\Delta$CDRSB) and MRI measurements ($\Delta$Hippocampus, $\Delta$WholeBrain, and $\Delta$Entorhinal) in the first year. For example, we defined the $\Delta$CDRSB as follows: $\Delta$CDRSB = (CDRSB_M12 − CDRSB_bl)/1 year (with “_M12” indicating the 12th month, with “_bl” indicating baseline). To compare the change rate in the first year (rate 1), the change rate in the second year (rate 2) and acceleration in the first 2 years (acceleration) were also computed.

**Table 4:** The prognosis models and the corresponding predictors for ADAS-13 (N = 110) and MMSE (N = 92) predictive dataset.

Prognosis models	Predictors
**Baseline**	
A1/M1	Age, gender, marital status, ApoEε4, ADAS-13_bl, MMSE_bl, and CDRSB_bl
A1+ baseline MRI/M1+ baseline MRI	A1/M1+ Hippocampus_bl, WholeBrain_bl, and Entorhinal_bl
**Longitudinal**	
A2/M2	Age, gender, marital status, ApoEε4, ADAS-13_bl, MMSE_bl, CDRSB_bl, $\Delta$ADAS-13, $\Delta$MMSE, and $\Delta$CDRSB
A2+ baseline MRI/M2+ baseline MRI	A2/M2+ Hippocampus_bl, WholeBrain_bl, and Entorhinal_blc
A2+ ∆MRI/M2+ ∆MRI	A2/M2+ $\Delta$Hippocampus, $\Delta$WholeBrain, and $\Delta$Entorhinal

A1, ADAS-13 baseline Prognosis Model; A2, ADAS-13 longitudinal Prognosis Model; M1, MMSE baseline Prognosis Model; M2, MMSE longitudinal Prognosis Model; ADAS-13_bl, Alzheimer Disease Assessment Scale Cognitive 13 items at Baseline; MMSE_bl, Mini mental state examination at Baseline; CDRSB_bl, Clinical Dementia Rating Sum of Boxes at Baseline; Hippocampus_bl, The Total Volumes of The Hippocampus at Baseline; WholeBrain_bl, Whole Brain at Baseline; Entorhinal_bl, Entorhinal Cortex at Baseline; ∆ADAS-13, (ADAS-13_M12 − ADAS-13_bl)/1 year; ∆MMSE, (MMSE_M12 − MMSE_bl)/1 year; ∆CDRSB, (CDRSB_M12 − CDRSB_bl)/1 year; ∆Hippocampus, (Hippocampus _M12 − Hippocampus _bl)/1 year; ∆WholeBrain, (WholeBrain _M12 − WholeBrain _bl)/1 year; ∆Entorhinal, (Entorhinal _M12 − Entorhinal _bl)/1 year.

The C-index was calculated for the comparison of different models, which is commonly used to evaluate the discriminative abilities of Cox models^37,38^. Additionally, we evaluated the improvements in discriminating ability attained with the extended models in comparison with the basic model by the net reclassification improvement (NRI; with cut = 10%)^37^, and the integrated discrimination improvement (IDI)^39–41^.

### Statistical analyses

The categorical variables in the present study were recorded as follows: gender (1: male, 2: female), marital status (1: married, 2: divorced, 3: others), education (1: medium (years of education < 12), 2: high (years of education ≥ 12)), and ApoEε4 (1: absent, 2: present).

Differences in demographic characteristics were tested using the chi-square test (for categorical variables), one-way analysis of variance (for continuous normally distributed variables), or the Kruskal–Wallis test (for continuous, non-normally distributed variables). X-tile software (version 3.6.1; Yale University School of Medicine, New Haven, CT, USA) was applied to the X-tile plots. An optimum cut-off was automatically selected by an approach provided by X-tile plots, and it was based on the highest chi-square statistic defined using the log-rank test and Kaplan–Meier survival analysis. To correct for optimism bias in the C-index, we undertook internal validation using 500 bootstrap samples^30,42,43^. All other statistical analyses were performed using SPSS 25.0 and R version 3.6.1. The R packages used in this study included survival, pec, and rms. GraphPad Prism 6 software was used to plot the data. A two-sided *P* < 0.05, was considered to indicate a significant difference.

## Supplementary Information

Supplementary Information.

## Data Availability

The data analyzed in the study are available from the ADNI website. (http://adni.loni.usc.edu).
