# Supplementary material for: Screening and predicting progression from high-risk mild cognitive impairment to Alzheimer’s disease
Source: Sci Rep. 2021 Sep 2;11:17558. doi: 10.1038/s41598-021-96914-3 (PMC8413294; doi:10.1038/s41598-021-96914-3)
Supplement: Supplementary file 1 — Supplementary Information. [file 41598_2021_96914_MOESM1_ESM.pdf]

Title Page

Screening and predicting progression from high-risk mild cognitive impairment to Alzheimer's disease

1.Xiao-Yan Ge[1,2]

[1]Department of Health Statistics, School of Public Health, Shanxi Medical University, 56 XinJian South Road, Taiyuan, China;

[2]Department of Health Statistics, School of Public Health, Jinzhou Medical University, 40 SongPo Road, Jinzhou, China.

2.Kai Cui

Department of Health Statistics, School of Public Health, Jinzhou Medical University, 40 SongPo Road, Jinzhou, China.

3.Long Liu

Department of Health Statistics, School of Public Health, Shanxi Medical University, 56 XinJian South Road, Taiyuan, China.

4. Yao Qin

Department of Health Statistics, School of Public Health, Shanxi Medical University, 56 XinJian South Road, Taiyuan, China.

5. Jing Cui

Department of Health Statistics, School of Public Health, Shanxi Medical University, 56 XinJian South Road, Taiyuan, China.

6. Hong-Juan Han

Department of Mathematics, School of Basic Medical Sciences, Shanxi Medical

University, Taiyuan, China.

7. Yan-Hong Luo

Department of Health Statistics, School of Public Health, Shanxi Medical University, 56 XinJian South Road, Taiyuan, China.

8. Hong-Mei Yu[1,2]

[1]Department of Health Statistics, School of Public Health, Shanxi Medical University, 56 XinJian South Road, Taiyuan, China;

[2]Shanxi Provincial Key Laboratory of Major Diseases Risk Assessment, 56 XinJian South Road, Taiyuan, China.

Corresponding author: Prof. HongMei Yu, Department of Health Statistics, School of Public Health, Shanxi Medical University, 56 XinJian South Road, Taiyuan, China. Tel.: +86 351 4135049, E-mail: yu@sxmu.edu.cn.

ORCID ID:yu@sxmu.edu.cn

Supplementary Table S1.

Hazard ratio (95%) of cox proportional hazards analysis based on ADAS-13 predictive dataset.

Predictors	A1	A1+baseline MRI	A2	A2+baseline MRI	A2+ΔMRI
Age	0.96 [0.93, 1.00]	0.95 [0.91, 0.99] *	0.96 [0.92, 1.00] *	0.95 [0.91, 1.00] *	0.92 [0.86, 0.98] *
Gender	1.20 [0.69, 2.08]	1.47 [0.65, 3.33]	0.86 [0.46, 1.59]	1.19 [0.49, 2.90]	0.68 [0.31, 1.49]
Marital status=2	0.45 [0.15, 1.34]	0.19 [0.05, 0.71] *	△	0.28 [0.07, 1.11]	0.24 [0.04, 1.34]
Marital status=3	0.82 [0.37, 1.83]	0.93 [0.42, 2.08]	△	1.09 [0.47, 2.68]	2.39 [0.68, 8.40]
ApoEε4	1.14 [0.66, 1.97]	1.69 [0.95, 3.00]	1.19 [0.67, 2.14]	1.19 [0.62, 2.29]	1.29 [0.60, 2.79]
ADAS-13_bl	1.06 [1.00, 1.12] *	1.14 [1.07, 1.21] ***	1.09 [1.02, 1.16] **	1.14 [1.06, 1.23] ***	1.13 [1.03, 1.24] **
MMSE_bl	0.89 [0.77, 1.04]	0.84 [0.71, 0.99] *	0.91 [0.76, 1.11]	0.88 [0.73, 1.07]	0.80 [0.62, 1.02]
CDRSB_bl	-	1.56 [1.23, 1.97] ***	2.05 [1.53, 2.72] ***	2.06 [1.53, 2.77] ***	1.70 [1.20, 2.40] **
CDRSB_bl1	△	-	-	-	-
Hippocampus_bl(cm^3)	-	0.83 [0.54, 1.28]	-	0.80 [0.52, 1.22]	-
WholeBrain_bl(cm^3)	-	1.00 [0.99, 1.01]	-	1.00 [0.99, 1.01]	-
Entorhinal_bl(cm^3)	-	0.90 [0.53, 1.51]	-	1.30 [0.75, 2.27]	-
ΔADAS-13	-	-	1.09 [1.03, 1.17] **	1.09 [1.00, 1.18] *	1.01 [0.91, 1.11]
ΔMMSE	-	-	0.95 [0.80, 1.12]	1.00 [0.84, 1.18]	0.81 [0.63, 1.03]
ΔCDRSB	-	-	2.08 [1.59, 2.72] ***	2.08 [1.54, 2.80] ***	2.41 [1.62, 3.58] ***
ΔHippocampus (cm^3)	-	-	-	-	1.91 [0.27, 13.4]
ΔWholeBrain (cm^3)	-	-	-	-	0.95 [0.91, 0.99] *
ΔEntorhinal(cm^3)	-	-	-	-	0.17 [0.04, 0.85] *

Notes. A1: ADAS-13 baseline Prognosis Model; A2: ADAS-13 longitudinal Prognosis Model; M1: MMSE baseline Prognosis Model; M2: MMSE longitudinal Prognosis Model. ADAS-13_bl, Alzheimer Disease Assessment Scale Cognitive 13 items at Baseline; MMSE_bl, Mini mental state examination at Baseline; CDRSB_bl, Clinical Dementia Rating Sum of Boxes at Baseline; ΔADAS-13, (ADAS-13_M12 – ADAS-13_bl) /1 year; ΔMMSE, (MMSE_M12 – MMSE_bl)

/1 year; ΔCDRSB, (CDRSB_M12 – CDRSB_bl) /1 year; Hippocampus_bl, The Total Volumes of The Hippocampus at Baseline; WholeBrain_bl, Whole Brain at Baseline; Entorhinal_bl, Entorhinal Cortex at Baseline; ΔHippocampus, (Hippocampus _M12 – Hippocampus _bl) /1 year; ΔWholeBrain, (WholeBrain _M12 – WholeBrain _bl) /1 year; ΔEntorhinal, (Entorhinal _M12 – Entorhinal _bl) /1 year. △: stratified variable. *p-value:* ***: $P < 0.001$; **: $P < 0.01$; *: $P < 0.05$.

Supplementary Table S2.

Hazard ratio (95%) of cox proportional hazards analysis based on MMSE predictive dataset.

Variables	M1	M1+baseline MRI	M2	M2+baseline MRI	M2+ΔMRI
Age	0.96 [0.92, 1.00]	0.93 [0.88, 0.98] **	0.96 [0.92, 1.01]	0.94 [0.88, 0.99] *	0.93 [0.86, 1.01]
Gender	0.90 [0.49, 1.67]	0.67 [0.26, 1.71]	0.97 [0.48, 1.93]	0.72 [0.28, 1.89]	0.55 [0.23, 1.34]
Marital status=2	0.80 [0.25, 2.50]	0.40 [0.10, 1.55]	0.61 [0.18, 2.02]	0.34 [0.08, 1.39]	0.21 [0.03, 1.30]
Marital status=3	1.13 [0.42, 3.03]	2.16 [0.77, 6.07]	0.72 [0.25, 2.06]	1.58 [0.53, 4.71]	1.33 [0.28, 6.30]
ApoEε4	0.97 [0.53, 1.78]	1.44 [0.76, 2.70]	1.01 [0.53, 1.92]	1.10 [0.54, 2.21]	0.99 [0.36, 2.70]
ADAS-13_bl	1.07 [1.02, 1.13] **	1.14 [1.07, 1.21] ***	1.10 [1.04, 1.17] **	1.16 [1.08, 1.24] ***	1.19 [1.07, 1.32] **
MMSE_bl	1.06 [0.89, 1.28]	0.94 [0.77, 1.16]	1.00 [0.80, 1.23]	1.00 [0.79, 1.26]	0.88 [0.65, 1.19]
CDRSB_bl	-	1.88 [1.39, 2.53] ***	-	2.07 [1.49, 2.89] ***	2.03 [1.33, 3.09] ***
CDRSB_bl1	△	-	△	-	-
Hippocampus_bl(cm^3)	-	0.82 [0.54, 1.24]	-	0.83 [0.53, 1.29]	-
WholeBrain_bl(cm^3)	-	1.00 [0.99, 1.01]	-	1.00 [0.99, 1.01]	-
Entorhinal_bl(cm^3)	-	0.87 [0.51, 1.49]	-	0.98 [0.56, 1.70]	-
ΔADAS-13	-	-	0.97 [0.79, 1.18] **	1.09 [1.00, 1.18] *	1.06 [0.96, 1.17]
ΔMMSE	-	-	1.10 [1.03, 1.17]	1.14 [0.92, 1.41]	1.11 [0.82, 1.48]
ΔCDRSB	-	-	1.66 [1.25, 2.21] ***	1.79 [1.30, 2.47] ***	2.26 [1.42, 3.60] ***
ΔHippocampus (cm^3)	-	-	-	-	1.43 [0.17, 12.0]
ΔWholeBrain (cm^3)	-	-	-	-	0.97 [0.92, 1.01]
ΔEntorhinal(cm^3)	-	-	-	-	0.29 [0.06, 1.47]

Notes. A1: ADAS-13 baseline Prognosis Model; A2: ADAS-13 longitudinal Prognosis Model; M1: MMSE baseline Prognosis Model; M2: MMSE longitudinal Prognosis Model. ADAS-13_bl, Alzheimer Disease Assessment Scale Cognitive 13 items at Baseline; MMSE_bl, Mini mental state examination at Baseline; CDRSB_bl, Clinical Dementia Rating Sum of Boxes at Baseline; ΔADAS-13, (ADAS-13_M12 – ADAS-13_bl) /1 year; ΔMMSE, (MMSE_M12 – MMSE_bl)

/1 year; ΔCDRSB, (CDRSB_M12 – CDRSB_bl) /1 year; Hippocampus_bl, The Total Volumes of The Hippocampus at Baseline; WholeBrain_bl, Whole Brain at Baseline; Entorhinal_bl, Entorhinal Cortex at Baseline; ΔHippocampus, (Hippocampus _M12 – Hippocampus _bl) /1 year; ΔWholeBrain, (WholeBrain _M12 – WholeBrain _bl) /1 year; ΔEntorhinal, (Entorhinal _M12 – Entorhinal _bl) /1 year. $\triangle$: stratified variable. *p-value:* ***: $P<0.001$; **: $P<0.01$; *: $P<0.05$.

Supplementary Table S3.

The performance of ADAS-13 and MMSE prognosis models validated by bootstrap, as indicted by Optimism-corrected concordance index (Optimism-corrected C-index) (B=500).

Model	Apparent	Bootstrap	Test	Optimism	Optimism. corrected	Optimism-corrected concordance index
A1	0.484	0.507	0.403	0.104	0.380	0.690
A1+baseline MRI	0.556	0.617	0.509	0.108	0.448	0.724
A2	0.650	0.689	0.618	0.071	0.579	0.790
A2+baseline MRI	0.701	0.621	0.544	0.078	0.623	0.812
A2+ΔMRI	0.772	0.799	0.686	0.113	0.659	0.830
M1	0.503	0.528	0.425	0.103	0.400	0.700
M1+baseline MRI	0.590	0.663	0.554	0.109	0.481	0.741
M2	0.602	0.652	0.549	0.102	0.500	0.750
M2+baseline MRI	0.704	0.677	0.578	0.100	0.604	0.802
M2+ΔMRI	0.725	0.748	0.606	0.142	0.583	0.792

Notes. A1: ADAS-13 baseline Prognosis Model; A2: ADAS-13 longitudinal Prognosis Model; M1: MMSE baseline Prognosis Model; M2: MMSE longitudinal Prognosis Model.
